# Elevated RIF1 participates in the epigenetic abnormalities of zygotes by regulating histone modifications on MuERV-L in obese mice

**DOI:** 10.1186/s10020-022-00446-z

**Published:** 2022-02-05

**Authors:** Jiliang Huang, Gaizhen Ru, Jiajia Sun, Luying Sun, Zhiling Li

**Affiliations:** Department of Reproductive Center, the First Affiliated Hospital of Shantou University Medical College, No. 57 Changping Road, Jinping District, Shantou, Guangdong 515041 People’s Republic of China

**Keywords:** RIF1, MuERV-L, Epigenetic modifications, Zygotic genome activation, Obesity

## Abstract

**Background:**

Maternal obesity impairs embryonic developmental potential and significantly increases the risks of metabolic disorders in offspring. However, the epigenetic transmission mechanism of maternal metabolic abnormalities is still poorly understood.

**Methods:**

We established an obesity model in female mice by high-fat diet (HFD) feeding. The effects of the HFD on the developmental potential of oocytes and embryos, the metabolic phenotype, and epigenetic modifications were investigated. The efficacy of metformin administration was assessed. Finally, the regulatory pathway of epigenetic remodeling during zygotic genome activation (ZGA) was explored.

**Results:**

Maternal HFD consumption significantly impaired glucose tolerance and increased the risk of metabolic disorders in F0 and F1 mice. Maternal HFD consumption also decreased embryonic developmental potential, increased reactive oxygen species (ROS) and γH2AX levels, and reduced the mitochondrial membrane potential (MMP) within oocytes, causing high levels of oxidative stress damage and DNA damage. Starting with this clue, we observed significantly increased RIF1 levels and shortened telomeres in obese mice. Moreover, significant abnormal DNA methylation and histone modification remodeling were observed during ZGA in obese mice, which may be coregulated by RIF1 and the ZGA marker gene MuERV-L. Metformin treatment reduced RIF1 levels, and partially improved ZGA activation status by rescuing epigenetic modification remodeling in oocytes and preimplantation embryos of obese mice. RIF1 knockdown experiments employing Trim-Away methods showed that RIF1 degradation altered the H3K4me3 and H3K9me3 enrichment and then triggered the MuERV-L transcriptional activation. Moreover, ChIP-seq data analysis of RIF1 knockouts also showed that RIF1 mediates the transcriptional regulation of MuERV-L by changing the enrichment of H3K4me3 and H3K9me3 rather than by altered DNA methylation.

**Conclusion:**

Elevated RIF1 in oocytes caused by maternal obesity may mediate abnormal embryonic epigenetic remodeling and increase metabolic risk in offspring by regulating histone modifications on MuERV-L, which can be partially rescued by metformin treatment.

**Supplementary Information:**

The online version contains supplementary material available at 10.1186/s10020-022-00446-z.

## Introduction

Obesity has become a global epidemic and a worldwide public health concern. A wide range of diseases caused by obesity seriously affect people’s health. Women of reproductive age are also challenged by obesity (Ng et al. 2014). Due to the pregnancy and offspring factors, more health problems and potential risks need to be considered in women of reproductive age. Obesity not only is associated with increased risks of almost every common complication of pregnancy, but also plays a direct role in the transgenerational transmission of an obesity or diabetes risk. Increasing evidence suggests that offspring of obese mothers are at increased risk of obesity (Bariani et al. 2020; Daxinger and Whitelaw 2012; Keleher et al. 2018), impaired glucose tolerance (Dunn and Bale 2009; Godfrey et al. 2017), and other facets of the metabolic syndrome (Catalano and Ehrenberg 2006; Volpato et al. 2012). The increased risks of metabolic abnormalities in offspring are probably associated with epigenetic abnormalities in maternal oocytes (Ou et al. 2019). However, it is not understood how these epigenetic abnormalities in maternal gametes are passed on to embryos, considering the extensive erasure and reconstruction of epigenetic modifications after zygotic genome activation (ZGA) (Eckersley-Maslin et al. 2018; Xia et al. 2019). Therefore, there is a growing need to understand how maternal diet influences embryo development and offspring health.

Endogenous retroviruses (ERVs) are important components of the mammalian genome (Mager and Stoye, 2015). Transcriptional activation of ERVs occurs during the transition from maternal control to zygotic genome control, signifying the onset of ZGA (Kigami et al. 2003). The murine endogenous retrovirus-like (MuERV-L) gene is an ERV3 family member, which accounts for 80% of recognized long terminal repeat (LTR) element copies predating the human-mouse speciation (Bénit et al. 1999). A significant number of 2-cell genes are initiated by the activation of MuERV-L (Macfarlan et al. 2012), suggesting that MuERV-L is one of the earliest transcribed genes in mouse one-cell embryos (Kigami et al. 2003). MuERV-L participates in rewiring gene expression networks during epigenetic reprogramming (Fu et al. 2019). MuERV-L can be silenced by various epigenetic regulators, including various material factors, histone H3 variants, H3K9 methyltransferases, and histone chaperones (Chen et al. 2020; Maksakova et al. 2013; Rowe et al. 2010). Therefore, we propose that MuERV-L can serve as an important checkpoint to study the epigenetic transgenerational mechanism during the maternal-to-zygotic transition (MZT).

Maternal factors may affect ZGA (Bultman et al. 2006; Wu and Dean 2020), mediated through MuERV-L. Which maternal factor plays a regulatory role under the influence of obesity is still a mystery. A recent study identified that Rap1-interacting factor 1 (RIF1) shows the strongest effect among a list of novel ERV regulators (Li et al. 2017). RIF1 was originally discovered as a factor involved in telomere length homeostasis (Hardy et al. 1992). The classical roles of RIF1 are related to the DNA damage response (Di Virgilio et al. 2013; Zimmermann et al. 2013), and DNA replication timing (Cornacchia et al. 2012; Yamazaki et al. 2012), indicating a relationship with oxidative stress. Studies have also indicated that RIF1 has important roles in embryonic stem cell (ESC) maintenance (Loh et al. 2006) and epigenetic gene regulation (Dan et al. 2014; Li et al. 2017). Emerging studies have highlighted the relationship between metabolic diseases and telomere length (Cheng et al. 2021; Vidacek et al. 2017), indicating that environmental stress affects the regulation of telomeres and RIF1. However, it has not been reported whether the RIF1 levels of oocytes are changed and further cause epigenetic abnormalities during ZGA in a model of obesity. Accordingly, we propose that RIF1 may be a maternal epigenetic regulator during ZGA in a model of obesity.

Despite the evidence above, the epigenetic transgenerational mechanism of metabolic-related abnormalities is not clear. Here, we attempted to explore the role of ERVs in participating epigenetic changes during the MZT of obese mice. Additionally, we investigated whether RIF1 is involved in regulating ERV activity and its epigenetic function.

## Materials and methods

### Animals, modeling, and protocols

Three- to four-week-old C57BL/6J mice were purchased from Hunan SJA Laboratory Animal Co., Ltd (Changsha, China) and were housed in the Laboratory Animal Center of Shantou University Medical College (conditions: a 12-h light/dark cycle, 21 ± 2 °C, SPF degree). All animal experiments were in accordance with the guidelines of the Medical Animal Care & Welfare Committee and were approved by the Laboratory Animal Ethics Committee of Shantou University Medical College (No. SUMC2020-383).

After adaptive feeding for 1 week, forty female mice were randomly divided into the high-fat diet (HFD) and control diet (CD) groups. Mice were fed either a HFD (H10060; HFK Bioscience, Beijing, China; containing 60% fat and 20% protein by energy ratio) or a control diet (provided by the Laboratory Animal Center of SUMC; containing 10% fat and 20% protein by energy ratio) for 8 weeks. Body weight was measured weekly during the 2 months.

In the research involving F1 generation mice, estrus or pro-estrus HFD and CD female mice were mated to CD male mice. The time of pregnancy was determined by visual inspection of the vaginal plug, which was defined as day 0 of pregnancy. Females were switched to CD at the time of mating. After weaning on day 21, offspring weight was recorded, and female pups were either fed a CD or HFD and weighed weekly (the CD and HFD compositions were the same as those fed to the mothers).

### Metformin intervention experiments

To clarify whether the epigenetic abnormalities induced by obesity could be reversed, metformin was used for a further intervention, according to the recommended doses in the previous studies (Abizadeh et al. 2020; Huang et al. 2015; Kamalipour et al. 2020). Briefly, 1000 mg of metformin was dissolved in 20 ml of saline and stored at 4 °C. Modeled mice were divided into the metformin and saline groups. Every mouse received oral gavage administration of 500 mg/kg/day metformin (No. D150959, Sigma, USA) or the corresponding normal saline (0.9% NaCl) for 20 consecutive days. Mice were weighed weekly to adjust the administered doses. After the completion of treatment, the mice were sacrificed for various subsequent experiments.

### Trim-Away degradation of RIF1 by electroporation

Trim-Away is a new method of degrading endogenous proteins by recruiting endogenous or exogenous TRIM21 to the antibody-bound target proteins (Clift et al. 2018). Protein degradation by Trim-Away is acute and rapid, with half-lives of ~ 10–20 min (Clift et al. 2017, 2018). To further test whether a decrease in RIF1 levels would induce subsequent epigenetic changes during ZGA, we employed an electroporation fashion to induce endogenous RIF1 protein reduction. In detail, metaphase II (MII) oocytes were incubated in the acidic Tyrode’s solution (T1788, Sigma, USA) to digest approximately 30% of the zona pellucida, which typically took 20–60 s. Subsequently, the oocytes were washed in M2 medium three times, and then pipetted into a 1-mm electroporation cuvette (Bio–Rad, catalog no. 1652089) with 20 µL Opti-MEM containing RIF1 antibody. The BioRad Gene Pulser XCell electroporator was used. Two square wave pulses were applied (voltage of 30 V, 5 ms pulse length, and 100 ms pulse interval) according to the published protocol (Maas et al. 2018). Immediately after electroporation, the oocytes were retrieved washed in M2 medium three times, and then cultured in KSOM (M1450; Aibei Biotechnology, China) for 1 h. Finally, the conventional in vitro fertilization procedures were applied.

### Glucose tolerance tests and insulin tolerance tests

For glucose tolerance tests (GTTs), 20% glucose (2 g/kg body weight) was fed by intragastrically administered after an overnight fasting. Blood glucose levels were measured using an Yuwell fasting glucometer (No.580, Yuyue Medical Equipment Co., Ltd, China). Blood was obtained from the tail vein, and glucose levels were monitored at 0, 15, 30, 60, 90, and 120 min after 2 months of feeding with a CD or HFD.

After one week of recovery from the GTTs, insulin tolerance tests (ITTs) were then applied in which mice were injected intraperitoneally with insulin (1 IU/kg body weight, Fosun Pharma Co., Ltd, China). Blood samples were collected and measured by the Yuwell fasting glucometer at 0, 15, 30, 60, 90 and 120 min after injection.

### Oocyte and embryo collection

To collect MII oocytes, female mice were superovulated by injecting 10 IU pregnant mare serum gonadotropin (PMSG) followed by 10 IU human chorionic gonadotropin (hCG) 48 h later and were sacrificed by cervical dislocation 14 h post-hCG injection (p-hCG). Cumulus–oocyte complexes (COCs) were released from the oviduct ampulla region. When fertilization was not required, COCs were transferred into medium containing 0.5 mg/mL hyaluronidase at 37 °C to digest cumulus granulosa cells for harvesting denuded MII oocytes.

For collecting embryos at each stage, in vitro fertilization and embryo culture were applied according to our previously published protocols (Huang et al. 2019). Briefly, spermatozoa, obtained from the cauda epididymis of CD male mice, were incubated in capacitation medium (HTF medium containing 1.5% BSA) at 37 °C in a 5% CO2 incubator for 1 h. Then, capacitated spermatozoa were added to a preprepared fertilization droplet (HTF medium containing 0.4% BSA) with COCs. After 6 h of fertilization, zygotes were either collected for subsequent experiments, or transferred into KSOM medium overlaid with mineral oil for collecting embryos at each stage.

### Reactive oxygen species and mitochondrial membrane potential

To detect the level of reactive oxygen species (ROS) and the mitochondrial membrane potential (MMP) in oocytes, dihydroethidium (S0063, Beyotime Biotechnology, Shanghai, China) and JC-1 kits (C2006, Beyotime Biotechnology, Shanghai, China) were applied respectively. According to our previous protocols (Li et al. 2019), a stock solution of dihydroethidium (1 × 10^−3^ mol/L in DMSO) was added to M2 medium to a final concentration of 10 μmol/L, and JC-1 was diluted in PBS to a final concentration of 1.25 μmol/L. Fluorescence staining was detected immediately under a fluorescence microscope (Nikon Eclipse 90 Ni-E). ImageJ (NIH Image, Bethesda, MD) was used to quantify the fluorescence intensity.

### Western blot

Western blotting was performed as described previously (Bariani et al. 2020; Han et al. 2018). A total of 100 MII oocytes or embryos were added to 2 × SDS sample buffer, incubated at 95 °C for 5 min and then frozen at -30 °C until further use. Protein samples were electrically separated by SDS–PAGE. Membranes were incubated at 4 °C overnight with the following primary antibodies: rabbit anti-PPARγ antibody (1:1000; CST, #2443), mouse anti-SIRT3 antibody (1:1000; Santa Cruz, sc-365175) or rabbit anti-GAPDH antibody (1:5000; Sigma, A5441). The appropriate species-specific horseradish peroxidase (HRP)-conjugated secondary antibodies (1:5000; Thermo Fisher Scientific, 31460 and 31430) were applied for a 1-h incubation at room temperature. Signals were measured using ECL western blotting substrate (4A Biotech, China) according to the manufacturer’s instructions.

### Quantitative real-time PCR

One hundred oocytes or embryos were collected from each group. RNA was extracted according to the manual of the Picopure™ RNA Isolation Kit (KIT0204, Thermo, USA). The concentration and quality of RNA were measured by a NanoDrop ND-2000 (Thermo, USA). cDNA synthesis was conducted using the PrimeScript First-Strand cDNA Synthesis Kit (6110A, Takara, Japan). Real-time PCR was performed with Takara RR420Q TB Green® Premix Ex Taq™ (RR420Q, Takara, Japan) in a Real-Time PCR Detection System (CFX96, Bio–Rad, USA). GAPDH was used as an internal control for each sample. The relative mRNA expression levels of target genes were calculated using the 2^−ΔΔCt^ method. Primers for qPCR were synthesized by GenePharma (GenePharma Co., Ltd, China) and are listed in Additional file 1: Table S1.

### Immunofluorescence (IF)

To detect a single target, denuded oocytes or embryos were digested with Tyrode’s solution to remove the zona pellucida, fixed with 4% paraformaldehyde for 30 min, and then permeabilized with PBS with 0.5% Triton X-100 for 20 min. After blocking in PBS containing 3% BSA and 10% fetal bovine serum for 1 h, samples were incubated with primary antibody at 4 °C overnight, followed by Alexa Fluor 555-conjugated goat anti-rabbit (1:200; ab150078, Abcam) or Alexa Fluor 488-conjugated goat anti-mouse (1:200; ab150113, Abcam) secondary antibodies at room temperature for 1 h. The primary antibodies used included RIF1 antibody (1:200; RayBiotech), MuERV-L Gag antibody (1:200; AF0240, Beyotime), H3K4me3 (1:200; ab1012, Abcam), H3K9me3 (1:200; Abcam), anti-γ-H2AX antibody (1:300; Abcam, ab22551), anti-PPARγ antibody (1:200; CST, #2443), and anti-SIRT3 antibody (1:100; Santa Cruz, sc-365175).

For detecting 5-methylcytosine (5mC) and 5-hydroxymethylcytosine (5hmC), immunofluorescence double staining was applied as described previously (Han et al. 2018). Briefly, an additional step of incubation in 3 M HCl solution for 30 min and neutralization in Tris–HCl (pH 8.0) for 10 min after fixation were administered, and the remaining procedures before primary antibody immunostaining were the same as those of a single target. After blocking, samples were stained overnight at 4 °C with rabbit polyclonal anti-5hmC antibody (1:200; 39769, Active Motif) and mouse monoclonal anti-5mC antibody (1:200; A-1014-010, Epigentek). The cells were washed and incubated with double secondary antibodies (1:200, ab150078 and ab150113, Abcam) for 1 h at room temperature.

After incubation with secondary antibodies, Antifade Mounting Medium containing DAPI (AAPR11-A5, China) was applied to cover the slides. Fluorescence staining was detected immediately under a fluorescence microscope (Nikon Eclipse 90 Ni-E). ImageJ (NIH Image, Bethesda, MD) was used to quantify the fluorescence intensity. To ensure comparability, identical conditions, including the same confocal microscope settings, were used for each group.

### Telomere measurement by quantitative real-time PCR

The relative telomere length (RTL) of oocytes was measured from the total genomic DNA using qPCR as previously described (Cheng et al. 2013). Briefly, 60 oocytes of each group were collected. DNA was extracted using the QIAmp DNA micro Kit (Qiagen 56304, Valencia, CA, USA). The telomere signal was normalized to the signal from the single-copy gene to generate a relative telomere to single-copy gene (T/S) ratio indicative of the RTL. A mouse 36B4 single-copy gene was used as the reference control gene. Equal amounts of DNA (300 pg) were used for each reaction. Primers for qPCR are listed in Additional file 1: Table S1.

### Quantification analysis of DNA methylation

The quantification of DNA methylation was validated as previously described (Pan et al. 2018; Urdinguio et al. 2015). Briefly, genomic DNA was extracted using the QIAmp DNA micro Kit (Qiagen 56304, Valencia, CA, USA). One hundred nanograms extracted DNA was loaded in each assay well. The global methylation of DNA was quantified using the Methylflash™ Global DNA Methylation (5‐mC) ELISA Easy Kit (#P-1030, Epigentek, USA) according to the manufacturer’s guidelines.

### Statistical analysis

Data are presented as the mean values ± SD. *P* < 0.05 was considered statistically significant. For statistical comparisons, ANOVA or Student’s t test was used in SPSS 17.0 software. GraphPad Prism 9.0 was used to draw cartograms. All experiments were repeated three times with a similar result, and representative results are shown in this article. ChIP-seq data were obtained from the GEO database. The Integrative Genomics Viewer (IGV) browser was used to display ChIP-seq data.

## Results

### Maternal HFD increased the risk of offspring metabolic disorder

**Fig. 1:**
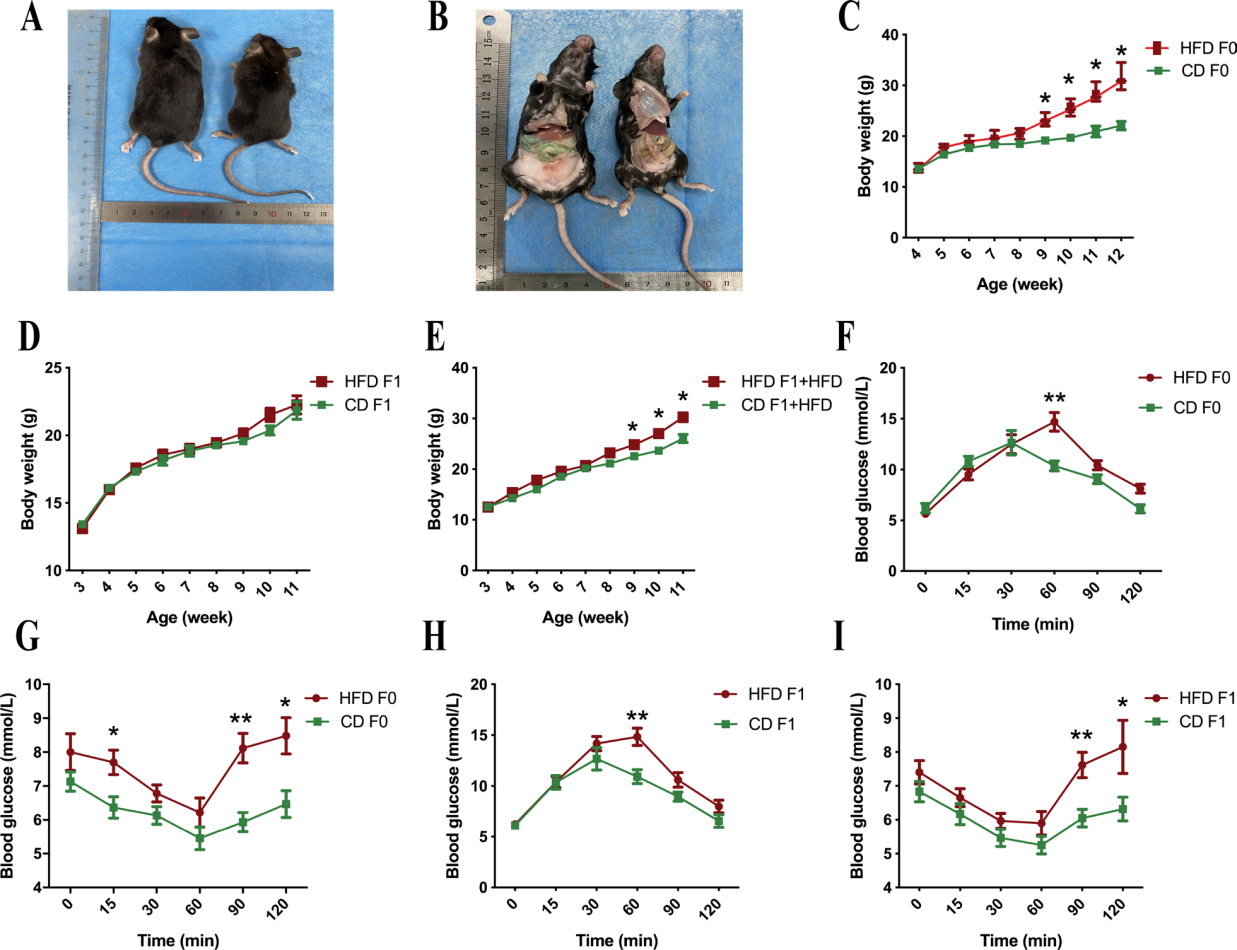
Effects of HFD on the metabolic phenotypes in F0 and F1 mice. **A**, **B** Anatomic features of the HFD and CD mice. **C** Weekly body weights of F0 mice between the HFD and CD groups (n = 6 for each group). **D** Weekly body weights of F1 mice fed the CD (n = 6 for each group). **E** Weekly body weights of F1 mice fed the HFD (n = 6 for each group). **F** Blood glucose levels of F0 mice between the HFD and CD groups when GTTs are applied (n = 5 for each group). **G** Blood glucose levels of F0 mice between the HFD and CD groups when ITTs are applied (n = 5 for each group). **H** Blood glucose levels of F1 mice between the HFD and CD groups when GTTs are applied (n = 5 for each group). **I** Blood glucose levels of F1 mice between the HFD and CD groups when ITTs are applied (n = 5 for each group). *p < 0.05, **p < 0.01. *HFD* high-fat diet, *CD* control diet

As shown in Fig. 1, an 8-week of HFD induced significant obesity, and increased the abdominal fat accumulation (Fig. 1A, B). We found that a significant difference in body weight emerged after one month of high-fat feeding between the groups (Fig. 1C). To understand whether a HFD leads to obesity in F1 offspring, we further compared the F1 body weight. The results showed that there was no significant difference in F1 body weight under CD (Fig. 1D). Furthermore, we show when a HFD was administered, we found that that the maternal HFD-fed F1 mice had a significantly higher weight than the maternal CD-fed F1 mice when the offspring receive postnatal HFD feeding (Fig. 1E). To determine the effect of a HFD on glucose metabolism, we conducted GTTs and ITTs. The results of the GTTs showed that higher blood glucose and delayed peak values appeared in the HFD-fed F0 mice (Fig. 1F, G) as well as in maternal HFD-fed F1 mice (Fig. 1H, I).

These results suggest that HFD feeding induces significant maternal obesity and impairs glucose tolerance in F0 and F1 mice. Maternal HFD feeding does not increase the body weight of F1 mice unless they receive postnatal HFD feeding.

### HFD leads to poor oocyte and preimplantation embryo results

#### Developmental damage

**Fig. 2:**
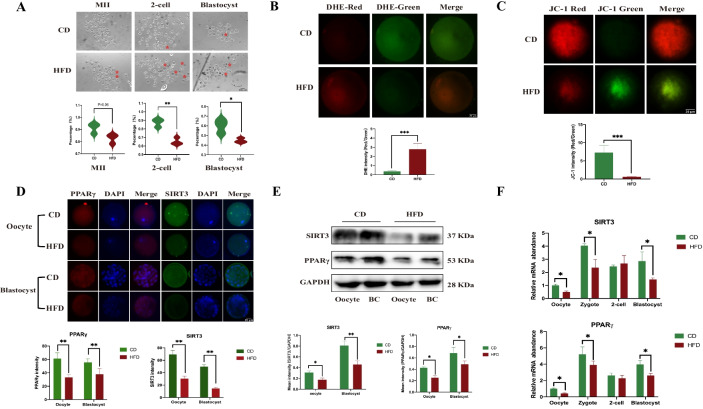
Development potential in oocytes and preimplantation embryos of HFD mice. **A** Developmental status captures of oocytes and embryos from the HFD and CD groups. Red asterisks indicate abnormal oocytes or embryos. Bottom: Statistical results of developmental status (oocytes, n = 90 for CD and n = 77 for HFD; 2-cell embryos, n = 80 for CD and n = 54 for HFD; blastocysts, n = 53 for CD and n = 23 for HFD). **B** Reactive oxygen species (ROS) of MII oocytes stained with dihydroethidium from the HFD and CD groups. The red fluorescence intensity represents the level of ROS. Bottom: The statistical results are shown between the HFD and CD groups. **C** Mitochondrial membrane potential (MMP) of MII oocytes stained with JC-1 from the HFD and CD groups. The ratios of the red/green fluorescence intensity indicated the levels of MMP, and Bottom: The statistical results are shown between the HFD and CD groups. **D** Representative immunofluorescence results of SIRT3 and PPARγ in oocytes and blastocysts from the HFD and CD groups. **E** Western blot analysis of SIRT3 and PPARγ in oocytes from the HFD and CD groups. F: qPCR results of SIRT3 and PPARγ from oocytes to blastocysts between the HFD and CD groups. Throughout, n = 20 in each group for IF analysis and n = 3 in each group for WB or qPCR analysis. Data are presented as means ± SD. *P* values are calculated by Student’s *t* test. *p < 0.05, **p < 0.01, ***p < 0.001. *ns* indicates not significant. Scale bars are shown in the lower right corner of the captures. *HFD* high-fat diet, *CD* control diet, *BC* blastocyst

The percentages of MII oocytes showed no significant difference between the HFD and CD groups (82.64 ± 3.94% vs. 91.33 ± 4.24%, *P* = 0.06) (Fig. 2A). However, the percentages of 2-cell and blastocyst formation were significantly lower in the HFD group than in the control group (65.29 ± 4.21% vs. 87.46 ± 4.85% and 44.76 ± 2.50% vs. 59.76 ± 7.16%, respectively; Fig. 2A), with more deaths or cytoplasmic fragmentation (red asterisk).

#### Oxidative stress

As shown in Fig. 2B, the fluorescence intensity ratios of DHE in MII oocytes from the HFD group (2.79 ± 0.61) were significantly higher than those of the CD group (0.37 ± 0.10, *P* < 0.001). Similarly, a JC-1 probe was used to detect MMP in oocytes, partly reflecting the functional status of the mitochondria. The results showed that the red/green fluorescence intensity ratios of JC-1 were significantly weaker in the HFD group (0.62 ± 0.11) than those in the CD group (7.298 ± 2.00,* P* < 0.001, Fig. 2C), indicating a decrease in MMP in the HFD group.

#### Metabolic function

PPARγ and SIRT3 were the most important candidates in the energy metabolic pathway (Gross et al. 2017; Wang et al. 2019). Therefore, the expression levels of PPARγ and SIRT3 in oocytes and preimplantation embryos were illustrated by IF, WB and qPCR. The IF results revealed that HFD caused deficiencies in PPARγ and SIRT3 levels in maternal oocytes (both *P* < 0.01, Fig. 2D), and these abnormalities in energy metabolism also occurred in the blastocysts (both *P* < 0.01, Fig. 2D), implying that they represent a precursor to metabolic abnormalities in offspring genes. As a validation of these findings, the results from WB and PCR also showed similar trends (Fig. 2E, F).

Accordingly, these results suggested that a HFD induced high levels of oxidative stress and impaired mitochondrial function and energy metabolism pathways in oocytes, which consistently persisted into the preimplantation embryos and ultimately led to an abnormal developmental potential of embryos and impaired energy metabolic function.

### Elevated RIF1 and epigenetic modification changes in oocytes and preimplantation embryos of obese mice

**Fig. 3:**
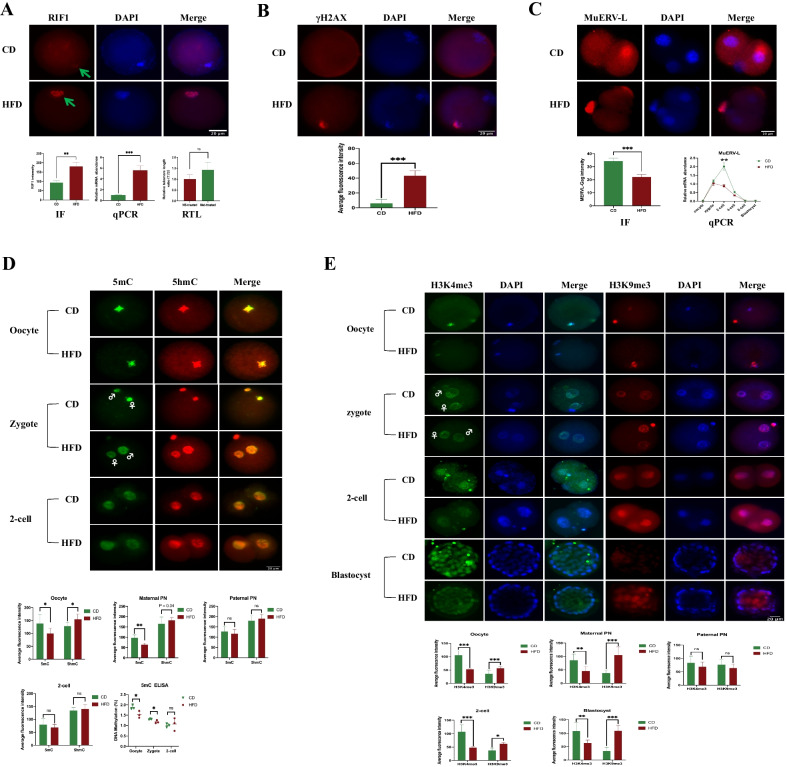
The effects of HFD on RIF1 and epigenetic modification changes in oocytes and preimplantation embryos. **A** The expression of RIF1 in oocytes of the HFD and CD groups. Top: representative immunofluorescence (IF) images. Arrowheads indicate the expression of RIF1 located in the nucleus. The bar chart in the middle of bottom: qPCR results of RIF1. The bar chart in the lower right corner: qPCR results of the relative telomere length (RTL). **B** Images of γ-H2AX staining in oocytes of the HFD and CD groups. Bottom: The statistical results are shown. **C** The expression of MuERV-L gag between the HFD and CD groups. Top: Representative IF images in 2-cell embryos. Since MuERV-L gag could hardly be detected at the other embryonic development stages, only images of 2-cell embryos are presented. Bottom: Statistical results from IF and qPCR. **D** IF images of 5mC and 5hmC enrichment during ZGA between the HFD and CD groups. The statistical diagrams and the ELISA results of 5mC are shown at the bottom. **E** IF images of H3K4me3 and H3K9me3 enrichment from oocytes to blastocysts between the HFD and CD groups. The statistical results are shown at the bottom. Throughout, n = 20 in each group for IF analysis, and n = 3 in each group for qPCR analysis. Data are presented as means ± SD. *P* values are calculated by Student’s *t* test. *p < 0.05, **p < 0.01, ***p < 0.001. *ns* indicates not significant. Scale bars are shown in the lower right corner of the captures. *HFD* high-fat diet, *CD* control diet. ♀ represents maternal pronuclear (PN). ♂ represents paternal PN

We explored whether the oxidative damage induced by HFD altered the levels of RIF1 and the RTL within the oocytes. We found that the expression of RIF1 was localized to the nucleus and that the average fluorescence intensity of RIF1 was significantly stronger in HFD oocytes than in CD oocytes (marked with green arrows, Fig. 3A). The qPCR results also confirmed a significantly higher expression of RIF1 in HFD oocytes (*P* < 0.001, the middle bar chart at the bottom of Fig. 3A). We inferred that the elevated RIF1 might be associated with the shortened telomeres and elevated γH2AX caused by the HFD. As shown in the right bar chart of Fig. 3A, the qPCR results indicated that the RTL in HFD oocytes was substantially shortened by 50%–60% compared with that in CD oocytes (*P* < 0.05). Additionally, γH2AX, a biomarker of DNA double-strand breaks, was significantly higher in the HFD group than in the CD group (*P* < 0.001, Fig. 3B). MuERV-L, an embryonic totipotency marker, was evaluated by IF and qPCR. IF staining showed that MuERV-L gag was detectable from the stage of 2-cell embryo stage (*P* < 0.001, Fig. 3C). Surprisingly, the qPCR results revealed that the expression of MuERV-L was most apparent in zygotes and 2-cell embryos (the right line chart at the bottom of Fig. 3C), which was quite different from the IF results. Regarding the changes in DNA methylation and histone modifications, we found that the average fluorescence intensities of 5mC in HFD oocytes and maternal pronuclear (PN) in HFD zygotes were significantly lower than those in the CD group, along with a significant increase in the 5hmC fluorescence intensities (Fig. 3D). However, no significant differences in either 5mC or 5hmC were found in the paternal PN region of zygotes and 2-cell embryos (all *P* > 0.05, Fig. 3D). Global DNA methylation from the oocyte to 2-cell embryo was validated by an ELISA-based colorimetric assay (the scatter diagram at the bottom of Fig. 3D). The data indicated that global 5mC in the HFD oocytes and zygotes was significantly lower than that in the CD group (1.53 ± 0.17 vs. 1.88 ± 0.11% and 1.17 ± 0.76 vs. 1.30 ± 0.24%, respectively; both *P* < 0.05), and no significant difference was observed in 2-cell embryo (1.07 ± 0.31% vs. 1.00 ± 0.09%, *P* = 0.73). For the histone modifications, the fluorescence intensities of H3K4me3 from oocytes to blastocysts (except for the paternal PN region of zygotes) in the HFD group were always significantly lower than those in the control group (all *P* < 0.05, Fig. 3E), whereas the opposite results were observed for H3K9me3 (all *P* < 0.05, Fig. 3E). Taken together, these results showed that obesity induced an elevated RIF1, the abnormal totipotency of zygotes, and the asymmetric epigenetic remodeling of both DNA methylation and histone modifications. These epigenetic abnormalities may be responsible for the increased risks of metabolic abnormalities in the offspring.

### Metformin treatment reduced RIF1 and modified epigenetic modification changes in oocytes and preimplantation embryos of obese mice

**Fig. 4:**
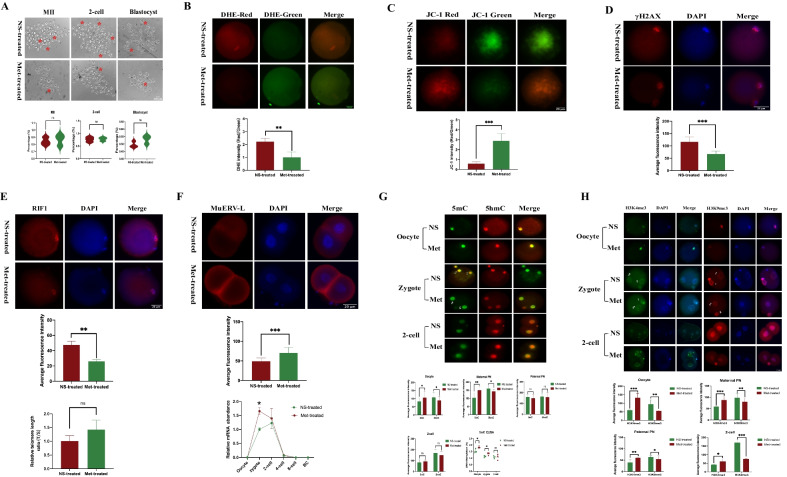
Metformin alleviated the adverse effects of HFD on oocytes and preimplantation embryos. **A** Developmental status captures of the oocytes and embryos in the NS-treated and Met-treated groups. Red asterisks indicate abnormal oocytes and embryos. Bottom: Statistical results of developmental status (oocytes, n = 121 for NS-treated group and n = 109 for Met-treated group; 2-cell embryos, n = 104 for NS-treated group and n = 92 for Met-treated group; blastocysts, n = 54 for NS-treated group and n = 51 for Met-treated group). **B** Reactive oxygen species (ROS) of MII oocytes stained with dihydroethidium (DHE) probes in the NS-treated and Met-treated groups. The red fluorescence intensity represents the level of ROS. Bottom: The statistical results are shown. **C** Mitochondrial membrane potential (MMP) of MII oocytes stained by JC-1 in the NS-treated and Met-treated groups. The ratios of the red/green fluorescence intensity indicated the levels of MMP. and Bottom: The statistical results are shown. **D** Images of γ-H2AX staining in oocytes of the NS-treated and Met-treated groups. Bottom: The statistical results are shown. **E** The expression of RIF1 in oocytes of the NS-treated and Met-treated groups. Top: Representative IF images. Middle: Statistical analysis results from IF. Bottom: qPCR results of the relative telomere length (RTL) of the NS-treated and Met-treated oocytes. **F** The expression of MuERV-L gag in the 2-cell embryos of the NS-treated and Met-treated groups. Bottom: Statistical results from IF and qPCR. **G** IF images of 5mC and 5hmC enrichment during ZGA between the NS-treated and Met-treated groups. The statistical diagrams and the ELISA results of 5mC are shown at the bottom. **H** IF images of H3K4me3 and H3K27me3 enrichment during ZGA between the NS-treated and Met-treated groups. Throughout, n = 20 in each group for IF analysis, and n = 3 in each group for qPCR analysis. Data are presented as means ± SD. *P* values are calculated by Student’s *t* test. *p < 0.05, **p < 0.01, ***p < 0.001. *ns* indicates not significant. Scale bars are shown in the lower right corner of the captures. NS-treated: normal saline-treated; Met-treated: metformin-treated. ♀ represents maternal pronuclear (PN). ♂ represents paternal PN

Unexpectedly, 20 days of metformin administration did not improve the MII rate of oocytes, 2-cell rate, or 8-cell rate (*P* = 0.73, *P* = 0.85 and *P* = 0.15, respectively; Fig. 4A). However, the disruptions of both ROS and MMP in oocytes were partially rescued (*P* < 0.01 and *P* < 0.001; Fig. 4B, C). As RIF1 was related to the DNA damage response, we evaluated the intensity of γH2AX again and found that metformin partially counteracted the elevated γH2AX caused by the HFD (*P* < 0.01, Fig. 4D). Additionally, metformin treatment significantly reduced RIF1 (top images and middle bar chart of Fig. 4E). However, no significant difference was found in the RTL (*P* = 0.14, bottom bar chart of Fig. 4E). The expression of MuERV-L detected by IF (top images and middle bar chart of Fig. 4F) and qPCR (bottom bar chart of Fig. 4F) all showed a significant amelioration, indicating an improvement in the embryo development potential by the administration of metformin. As shown in Fig. 4G, the loss of DNA methylation in the oocytes and in the maternal PN region of HFD zygotes were both significantly improved (*P* = 0.02 and *P* < 0.001, respectively) when metformin was administered. Correspondingly, the 5hmC in the oocytes and the maternal PN region of HFD zygotes were significantly reduced (*P* = 0.04 and *P* < 0.001, respectively). Similar to the IF results, the ELISA results of global 5mC indicated that significant increases in DNA methylation occurred in the Met-treated oocytes and zygotes than those of NS-treated group (both *P* < 0.05, the scatter diagram at the bottom of Fig. 4G). In contrast to the variation tendency of DNA methylation, abnormalities in histone modifications, including H3K4me3 and H3K9me3, were significantly modified from the oocytes to the blastocyst embryos (all *P* < 0.05, Fig. 4H). In other words, the impact of metformin intervention had a stronger effect on histone modifications than on DNA methylation.

Taken together, the findings above indicated that metformin administration was associated with some improvements in oocyte antioxidant activity, DNA damage, ZGA and epigenetic remodeling, although no significant improvements in the blastocyst formation rate were observed.

### Knockdown of RIF1 changes the totipotent ability and epigenetic modification of zygotes during ZGA

**Fig. 5:**
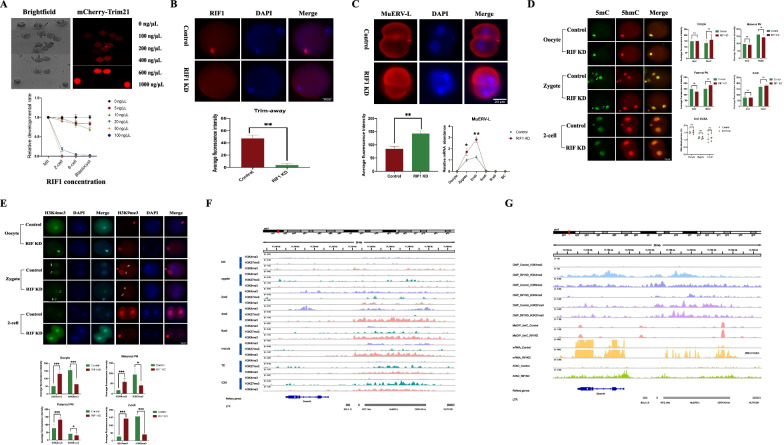
RIF1 regulates MuERV-L interaction with H3K4me3 and H3k9me3. **A** Various concentrations of mCherry-Trim21 mRNA and RIF1 antibody were evaluated with the developmental abilities of embryos. Top: Various concentrations of mCherry-Trim21 mRNA. Bottom: Various concentrations of RIF1 antibody. **B** The remaining protein intensities of endogenous RIF1 were obtained between the RIF1 KD and control groups when the 10 ng/μL RIF1 antibody was electroporated into the oocytes. Bottom: Statistical results are shown. **C** The expression of MuERV-L in the 2-cell embryos of the RIF1 KD and control groups. Top: Representative immunofluorescence (IF) images of 2-cell embryos. Bottom: Statistical results from IF and qPCR. **D** IF images of 5mC and 5hmC enrichment during ZGA between the RIF1 KD and control groups. The statistical diagrams and the ELISA results of 5mC are shown at the bottom. **E** IF images of H3K4me3 and H3K9me3 enrichment during ZGA between the RIF1 KD and control groups. **F** ChIP-seq track signals of H3K4me3, H3K9me3 and H3K27me3 from mouse oocytes to blastocysts, which were extracted from GSE73952 and GSE 98149, and visualized by Integrative Genomics Viewer (IGV). **G** Track signals of H3K4me3, H3K9me3, H3K27me3, 5mC, and mRNA expression at the MuERV-L-int locus in control and RIF1 KD ESCs, which were extracted from GSE98356. Throughout, n=20 in each group for IF analysis, and n = 3 in each group for qPCR analysis. Data are presented as means ± SD. *P* values are calculated by Student’s *t* test. *p < 0.05, **p < 0.01, ***p < 0.001. Scale bars are shown in the lower right corner of the captures. RIF1 KD: RIF1 knockdown. ♀ represents maternal pronuclear (PN). ♂ represents paternal PN

To clarify whether the elevated RIF1 in HFD oocytes mediated the developmental abnormalities and epigenetic remodeling in the embryos, we performed knockdown experiments by electroporating an anti-RIF1 antibody into oocytes, which induced a transient decrease in RIF1 protein levels based on the Trim-Away principle by binding Trim21. First, the effectiveness of electroporation was validated by mCherry-Trim21 mRNA, which allows visualization of Trim21 in living cells via red fluorescence. Visible fluorescent mCherry-Trim21 protein could be captured when mCherry-Trim21 mRNA was added to the electroporation reagents (Fig. 5A). Next, various concentrations of Trim21 and RIF1 antibodies were tested (Fig. 5A). However, we found that when cotransferring exogenous Trim21 and RIF1 antibodies into the oocytes, it would lead to a persistent deficiency of endogenous RIF1, which ultimately led to embryonic arrest. We even used mCherry-Trim21 mRNA at a concentration of 100 ng/μL, which was already lower than the reported concentration (Gerri et al. 2020). Therefore, only RIF1 antibody was electroporated into the cell, and endogenous RIF1 was slightly knocked down by endogenous Trim21. Finally, 10 ng/μL of RIF1 antibody was selected as an effective concentration, which not only ensured continued embryo survival but also significantly reduced endogenous RIF1 (line chart at the bottom of Fig. 5A).

The functional consequences were assessed using the concentrations outlined above. We confirmed that the protein level of endogenous RIF1 was reduced when 10 ng/μL RIF1 antibody was electroporated into oocytes (*P* < 0.01, Fig. 5B). Moreover, the effects of RIF1 knockdown on ZGA were evaluated. As shown in Fig. 5C, knockdown of RIF1 elevated the expression level of MuERV-L, as validated by both IF and qPCR. Unexpectedly, both the IF and ELISA results of 5mC indicated that the knockdown of RIF1 did not alter DNA methylation levels from the oocytes to 2-cell embryos in HFD mice (all *P* > 0.05, Fig. 5D). However, global increased enrichment of H3K4me3 and decreased enrichment of H3K9me3 were also observed in the maternal PN region of RIF1 knockdown zygotes by IF (Fig. 5E). To further clarify the enrichment changes in the histone modifications of the promoter region of MuERV-L, the ChIP-seq data in recent public datasets (GSE73952, GSE98149, and GSE98256) were analyzed. We first examined the common histone modifications at all stages from oocytes to blastocysts and found that the enrichment of H3K27me3 on MuERV-L was not strong from during ZGA (Fig. 5F). That is, the H3K4me3 and H3K9me3 histone modifications play a greater role than H3K27me3 in the regulating the transcription of MuERV-L during ZGA. This observation was also consistent with the results of our main investigations of H3K4me3 and H3K9me3 in this study. Furthermore, the knockdown of RIF1 did not cause changes in H3K27me3 enrichment on MuERV-L, but rather caused significant changes in H3K4me3 and H3K9me3 enrichment (Fig. 5G), which was highly consistent with the results obtained by Trim-Away (Fig. 5E). ATAC-seq showed that the knockdown of RIF1 significantly increased mRNA transcription and genome accessibility around RIF1-bound ERV loci (Fig. 5G), indicating enhanced activation of ZGA.

Altogether, these data suggested that RIF1 mediated the assembly of histone modifications on MuERV-L and that elevated RIF1 induced by maternal obesity could facilitate ZGA transcriptional activity and epigenetic remodeling by regulating the histone modifications of MuERV-L.

## Discussion

**Fig. 6:**
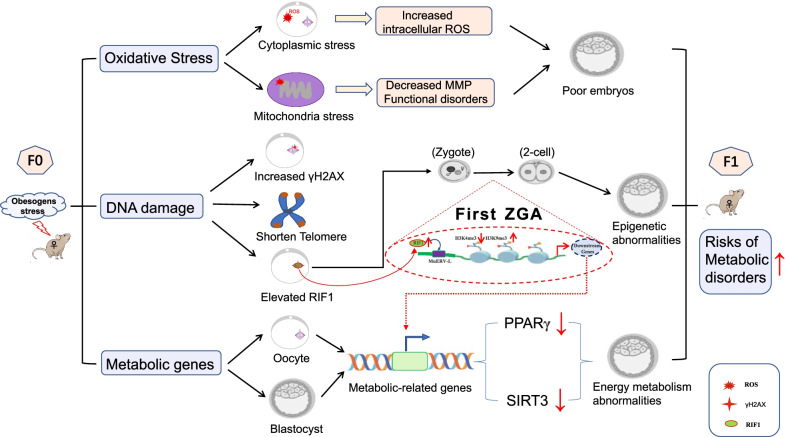
Diagram summarizing the influencing factors of offspring phenotype and the proposed epigenetic mechanism in a HFD mouse model

In this study, we conducted a comprehensive and detailed phenotypic assessment of oocytes and preimplantation embryos induced by the maternal obesity, and identified that RIF1 could act as a maternal epigenetic factor related to DNA stress damage and that its overexpression can mediate metabolic phenotypic differences by regulating epigenetic modifications on MuERV-L in obese mice, which could be partially rescued by metformin treatment. A schematic diagram of this mechanism is shown in Fig. 6.

ZGA is controlled by maternal factors, including protein and RNA stored in oocytes (Lee et al. 2014; Wu and Dean 2020), which are influenced by the prepregnancy environment, aging factors, oxidative stress, and lifestyle factors. Different levels of maternal proteins, acting as epigenetic factors or pioneer transcription factors, may initiate ZGA by inducing the opening of chromatin and recruiting other transcriptional machinery (Lee et al. 2014). Our results showed that RIF1 levels were significantly elevated in oocytes of HFD mice. RIF1, as a negative regulator of telomere elongation (Hardy et al. 1992), is involved in the DNA oxidative damage response (Di Virgilio et al. 2013; Zimmermann et al. 2013). As a validation, we found that HFD induced elevated ROS and γH2AX, and impaired mitochondrial function, and reduced RTL in oocytes. A previous study also showed colocalization of RIF1 with γH2AX in the DNA damage sites (Renaud et al. 2016). It has been reported that RIF1 interacts with H3K9 methylation to mediate heterochromatic silencing, which indicates its roles in epigenetic gene regulation (Dan et al. 2014). Similar findings in our results showed that HFD feeding caused elevated RIF1, accompanied by decreased expression of MuERV-L and impaired establishment of epigenetic asymmetry including DNA methylation and histone modifications. Therefore, RIF1, as an obesity-associated maternal factor, may be involved in epigenetic remodeling.

To further support the main role of RIF1 in regulating ZGA, the Trim-Away experiments and Chip-seq data in this study illustrated that RIF1 engaged in modulating the activation state of MuERV-L by interacting with and facilitating H3K4me3 and H3K9me3 but not DNA methylation. Knockdown of RIF1 further caused the transcriptional activation of MuERV-L and the remodeling of histone modifications. It is worth noting that knockout of RIF1 leads to failure in embryonic developmental (Buonomo et al. 2009). Therefore, ensuring further developmental potential is critical when endogenous RIF1 is degraded. Trim-Away, as a recent technique, degrades endogenous proteins guided by antibodies binding with endogenous or exogenous TRIM21 (Clift et al. 2017). Unlike DNA- or RNA-targeting methods, which take hours or days to deplete proteins of interest, the Trim-Away system removes endogenous proteins within minutes (Di Virgilio et al. 2013). Since the initial activation of ZGA occurs within a few hours after fertilization, we proposed that Trim-Away was the best method to study transient protein depletion during ZGA. Consistent with our study, other researchers have also reduced endogenous transcription factors in mouse zygotes and embryos using the Trim-Away assay (Gerri et al. 2020; Israel et al. 2019; Mehlmann et al. 2019). It is demonstrated that RIF1 not only facilitates H3K9me3 and H3K27me3 occupancy at ERVs but also interacts with histone marks (Vermeulen et al. 2010), either directly or indirectly via Chaf1a. RIF1 knockout causes global alterations of compartments and epigenetic states (Klein et al. 2021), showing the strongest effect among a list of novel ERV regulators (Li et al. 2017). However, these results were investigations at the embryonic stem cell level, which did not fully represent the changes in zygotes. Our study precisely illustrated the role of RIF1 in the mouse zygote. Hence, it is conceivable that RIF1 is an essential factor during ZGA but that abnormally elevated RIF1 caused by DNA damage will affect the transcriptional activation of MuERV-L and the deposition of histone modifications on MuERV-L.

In this study, we identified that MuERV-L, as an acting target element of RIF1, began at 6 hpi at the 1-cell stage and reached its peak at the 2-cell stage. The activation state of MuERV-L also significantly affected the genome-wide enrichment of epigenetic remodeling. These results sufficiently suggested that MuERV-L appeared to be an important regulatory element of epigenetic modifications. Concordant with our results, MuERV-L has been reported to be highly expressed in 2-cell embryos and to decline sharply at the later stages (Kigami et al. 2003), showing a close relationship with open chromatin (Wu et al. 2016). Chromatin accessibility is important for the control of selective gene expression. MuERV-L is one of the earliest transcribed genes in mouse 1-cell embryos (Kigami et al. 2003) and promotes the expression of hundreds of neighboring genes (Schoorlemmer et al. 2014). Other studies showed that the expression of MuERV-L was regulated by some maternal factors such as REX1 (Schoorlemmer et al. 2014), TRPS1 (Liu et al. 2019), and DPPA3 (Huang et al. 2017), contributing to the epigenetic plasticity of mouse embryos. As H3K9me3 is known to be required for silencing ERVs (Elsässer et al. 2015; Matsui et al. 2010), Didier Trono’s group demonstrated that SETDB1 regulates the deposition of H3K9me3 and controls the activation of MuERV-L by interacting with KAP1 in ESCs (Rowe et al. 2010). However, our study found that RIF1 was also involved in regulating the transmission of the embryonic metabolic phenotype by regulating MuERV-L, interacting with the deposition of H3K4me3 and H3K9me3. This fnding is consistent with the classical modification pattern of heterochromatin (Groh and Schotta 2017). Accordingly, we propose that RIF1 is an important regulatory element that mediates abnormal epigenetic reconstruction in obese mice.

Obesity is a worldwide health problem, involving multiple abnormal phenotypes such as inflammation, oxidative stress, DNA damage, and mitochondrial dysfunction in oocytes (Andreas et al. 2019; Snider and Wood 2019). Obesity, which is correlated with an inactive lifestyle, is associated with short telomeres (Cheng et al. 2021). Our results showed that metformin administration significantly reduced RIF1 and improved ZGA status and epigenetic remodeling but did not improve telomere length. Metformin, as an oral insulin-sensitizing agent, is widely used in the treatment of patients with type II diabetes, insulin resistance, metabolic syndrome and polycystic ovary syndrome. Previously published studies also summarized the effects of metformin on the epigenome, including epigenetic-modifying enzymes, histone and DNA methylation, and gene expression (Bridgeman et al. 2018; Faure et al. 2018). Consistent with our findings, a recent study showed that metformin could reduce oxidative stress and H3K9me3 in the oocytes of PCOS (Amani Abkenari et al. 2021). Unlike the Trim-Away results of RIF1, the metformin intervention improved the DNA methylation status in addition to improving the histone modification. As a possible mechanism involved in these epigenetics, metformin treatment may increase DNA methylation via its effect on one-carbon metabolism (Cuyàs et al. 2018).

Our results indicate that maternal HFD causes epigenetic changes and that these abnormalities can be transmitted to offspring. Our results are consistent with a previous report that environmental stress can reprogram the epigenome of the germline (sperm and eggs), which transmits the susceptibility to disease to future generations through epigenetic transgenerational inheritance (King and Skinner 2020). However, it remains controversial whether parental non-genetic modifications could be consistently transmitted to offspring. A recent study from Anne C. Ferguson-Smith's group showed common reprogramming of methylation states after fertilization, indicating that memory of the parental methylation state is an exception rather than the rule (Kazachenka et al. 2018). In our opinion, the environment-induced epigenetic variations can partly be retained during the ZGA, but these variations may not be stably existed. The original epigenetic changes may be erasured or disappeared and newly epigenetic imprintings will be rewritten and established with the continuous environmental stress. A similar supporting study found that the ART-associated epigenetic variation during embryo culturing would be largely resolved by adulthood (Novakovic et al. 2019). Therefore, we believe that our findings are not contradictory to the viewpoints of Anne C. Ferguson-Smith's study. First, the study of Anne C. Ferguson-Smith's group investigated only DNA methylation. However, our results suggest that histone modifications on MuERV-L play a more important epigenetic role than DNA methylation. Second, although the histone modifications will be extensively erasured and rebooted, low fold enrichment of noncanonical H3K4me3 markers still exists during ZGA (Liu et al. 2018; Xia et al. 2019), indicating that histone modifications may be a possible mechanism for mediating epigenetic intergenerational transmission. Third, the authors tested only six novel VM-IAPs, and one VM-IAP (Gm13849), showed a non-genetic inheritance effect. Their results do not refute the legitimacy of classic paradigms of non-genetic inheritance, as noted by Professor Darren J. Burgess (Kazachenka et al. 2018). Fourth, we infer that the epigenetic imprinting is metastable and can be continuously erasured and rewritten. This viewpoint is consistent with the viewpoints of Anne C. Ferguson-Smith's group.

## Limitations

Some limitations should be addressed in this study. First, a single dose of metformin was used in our study. Although a dose of 500 mg/kg was used in some studies and proved to be useful for improving embryonic development, whether there is dose-dependent improvement in epigenetic remodeling by metformin, the relationship between the dose of metformin and RIF1 level and the level at which RIF1 should be controlled still need to be determined, considering that RIF1 depletion leads to early embryonic lethality. Second, due to the difficulty in collecting amounts of preimplantation embryos for histone sequencing experiments (CUT&Tag or ChIP-seq), the histone sequencing data were obtained from the GEO database, which was another major limitation. Finally, it was confirmed in this study that RIF1, as a maternal epigenetic factor, was involved in regulating the epigenetic remodeling of ERVs during ZGA, but whether there are other factors that are jointly involved in this process requires further investigation.

## Conclusion

We concluded, for the first time, that maternal obesity caused an increase in RIF1 levels in oocytes, resulting in reduced embryonic developmental potential and epigenetic asymmetry remodeling, which in turn led to an impaired metabolic phenotype of offspring. RIF1 may be a key point for the treatment of epigenetic abnormalities. Indeed, metformin treatment is beneficial for preventing epigenetic transmission of maternal metabolic abnormalities.

## Supplementary Information

**Additional file 1: Table S1**. Quantitative real-time PCR primers used in this study.

## Data Availability

The main original data are provided in this manuscript and supplementary files. Any other original data are available from the corresponding author when needed. The ChIP-seq datasets analyzed in this study were previously published and available at the GEO website (GSE73952, GSE98149, GSE98256).

## Abbreviations

5mC: 5-Methylcytosine
5hmC: 5-Hydroxymethylcytosine
CD: Control diet
COCs: Cumulus–oocyte complexes
ERVs: Endogenous retroviruses
ESC: Embryonic stem cell
GTTs: Glucose tolerance tests
hCG: Human chorionic gonadotropin
HFD: High-fat diet
IF: Immunofluorescence
IGV: Integrative Genomics Viewer
ITTs: Insulin tolerance tests
MII: Metaphase II
MMP: Mitochondrial membrane potential
MuERV-L: Murine endogenous retrovirus-like
MZT: Maternal-to-zygotic transition
RIF1: Rap1-interacting factor 1
PMSG: Pregnant mare serum gonadotropin
ROS: Reactive oxygen species
RTL: Relative telomere length
ZGA: Zygotic genome activation
